# Eating disorders and disordered eating behaviors in the LGBT population: a review of the literature

**DOI:** 10.1186/s40337-020-00327-y

**Published:** 2020-10-16

**Authors:** Lacie L. Parker, Jennifer A. Harriger

**Affiliations:** 1 Department of Psychology, Loma Linda University, 11130 Anderson Street, Suite 106, Loma Linda, CA 92350 USA; 2 Pepperdine University, Malibu, USA

**Keywords:** Eating disorders, Disordered eating behaviors, LGBT, Sexual minority, Lesbian, Gay, Bisexual, Transgender

## Abstract

**Background:**

According to past research, lesbian, gay, bisexual, and transgender (LGBT) individuals experience a higher prevalence of psychopathology, which is attributable to the increased stress (i.e., stigma and prejudice) that they experience, as detailed by the minority stress model (MSM).

**Main:**

This current literature review examined the empirical literature regarding the rates and types of, and risk factors for eating disorders and disordered eating behaviors in LGBT adults and adolescents, in addition to each individual subgroup (i.e., lesbians, gay males, bisexuals, transgender and gender-nonconforming individuals).

**Conclusion:**

LGBT adults and adolescents experience greater incidence of eating disorders and disordered eating behaviors than their heterosexual and cisgender counterparts. Additionally, gay, bisexual, and transgender adults and adolescents were all at increased risk for eating disorders and disordered eating behaviors. Mixed results were found for lesbian adults and adolescents. Results are discussed within the framework of the MSM.

## Plain English summary

It has been found that lesbian, gay, bisexual, and transgender (LGBT) adults and adolescents are more likely to suffer from mental illness due to experiencing greater stress, caused by stigma and prejudice. This literature review examines past research findings regarding eating disorders and disordered eating behaviors for lesbian, gay, bisexual, transgender and gender non-conforming adults and adolescents as a whole, as well as each individual group. Overall, it was found that lesbian, gay, bisexual, and transgender adults and adolescents are more likely to experience eating disorders and disordered eating behaviors. Additionally, unique risk factors were identified for each lesbian, gay, bisexual, and transgender adult and adolescent group.

## Background

While eating disorders and disordered eating behaviors can affect individuals with various identities, it has been found that disparities exist in certain marginalized groups, such as sexual and gender minorities [145]. The purpose of this research is to review the literature regarding eating disorders and disordered eating behaviors within lesbian, gay, bisexual, and transgender (LGBT) adults and adolescents in comparison to their heterosexual and cisgender counterparts. Additionally, we examined four specific LGBT subgroups (lesbian adults and adolescents; gay adults and adolescents; bisexual, mostly heterosexual, and questioning adults and adolescents; transgender and gender non-conforming adults and adolescents), as well as risk factors for each subgroup.

The minority stress model (MSM) is often used to explain mental health disparities in sexual [121] and gender minority [76] groups. Minority stress models posit that individuals from LGBT populations experience unique distal stressors, such as stigma and discrimination, and proximal stressors, such as internalized homophobia or transphobia and concealment of sexual or gender identity [122] which in turn lead to increased risk for the development of physical and mental health issues [26, 101, 118, 121, 122]. For example, one study found that sexual minority adolescents reported various forms of stress from the original model [121] including distal (discrimination and victimization), proximal (expectations of rejection and internalized stigma such as homophobia), and disclosure (concealment stress), as well as violence and social and verbal victimization [59]. It has also been found that sexual minority youth report higher levels of sexual minority-specific victimization, depressive symptoms, and suicidality compared to their heterosexual peers [30]. Additionally, research has found that individuals from sexual and gender minority groups that perceive higher levels of stigma are more likely to report eating disorder symptoms [14, 110], and that shame, concealment of one’s sexual identity, and discrimination increases the risk of eating disorders in sexual minority men and women [12, 163, 166]. Finally, minority stress is related to binge-eating in lesbian and bisexual women [106] and body dissatisfaction in gay males [94]. While minority stress is only one of the frameworks from which to understand eating disorder risk in sexual and gender minorities, it is important to examine research on LGBT individuals through the lens of minority stress.

Indeed, it has been reported that both clinical eating disorders and eating disorder behaviors occur more frequently in LGBT individuals compared to their heterosexual and cisgender counterparts [10, 32, 44, 73, 145, 160]. Further research has indicated that approximately 54% of LGBT adolescents have been diagnosed with a full-syndrome eating disorder during their lifetime, with an additional 21% suspecting that they had an eating disorder at some point during their life [157]. Additionally, 60.9% of LGBT adolescents in one study reported engaging in at least one disordered eating behavior within the past year [61]. Transgender youth were more likely to report eating disorder behaviors compared to cisgender populations [44, 66, 169]. Finally, a recent study examining participants upon admission to eating disorder treatment reported that sexual and gender minority participants had more acute eating disorder symptoms and higher rates of abuse compared to cisgender heterosexual participants [119].

Adult sexual minorities have been found to have experienced significant disordered eating symptomology, including desire to be thin, bingeing, purging, and body dissatisfaction, which correlated with being overly concerned about body shape and size and level of femininity (regardless of sex assigned at birth). These behaviors occurred at higher rates than within the heterosexual and cisgender male population, but did not appear to be significantly different from heterosexual and cisgender females [37, 44, 120, 145, 151]. Adult sexual minorities also were almost twice as likely to experience food addiction (defined as the application of the criteria of substance use disorder to highly rewarding foods) compared to heterosexuals, which was aggravated by heterosexist harassment [135], and sexual minorities have reported higher rates of weight discrimination than heterosexual peers, even after controlling for body mass index (BMI) and race [145]. Other studies indicated that LGBT youth engaged in disordered eating behaviors, such as purging, fasting, dieting with intention of weight loss, and taking diet pills at higher rates compared to heterosexual youth [5, 67, 84, 113, 154, 167], putting them at greater risk for developing an eating disorder [31]. Further, the results of one study indicated that over half of sexual and gender minority youth experienced weight-based victimization from family members and peers, which in turn was associated with increased rates of binge eating, dieting, and other unhealthy weight-control behaviors, in addition to stress, exercise avoidance, less physical activity, and poorer sleep [79].

Gordon et al. [61] also found that their young adult and adolescent participants indicated that their appearance ideals came from traditional media, social media, LGBT-specific media, dating apps, and family, suggesting that LGBT appearance ideals only slightly differs from heterosexual and cisgender appearance ideals, with the exception of the influence of LGBT-specific media. Four themes emerged regarding the participants’ experiences with these appearance ideals: (1) appearance ideals tended to interrelate with one’s sexual and/or gender identity development, in that respondents constructed their own ideal from a wide variety of sources, especially from sources that aligned with their own sexual and/or gender identity; (2) appearance ideals and LGBT stereotypes were intertwined, in that others expected them to have a certain appearance based on the stereotype of their sexual and/or gender identity (e.g., gay men stereotypically have a lean and muscular body); (3) gender identity, sexual orientation, and race/ethnicity all uniquely contributed to the pressure one felt to appear a certain way; and (4) LGBT-specific community spaces had the potential to be either affirming or constraining to one’s appearance, in that other sexual and gender minorities were either accepting of a variety of body shapes and sizes, or reinforced societal expectations of the ideal body type.

Other research findings suggest that the sexual minority community has both protective and detrimental effects on adult LGB individuals’ body image and eating behaviors. For example, a qualitative study of LGB adults conducted by VanKim et al. [160] found that while many participants reported that their sexual orientation motivated them to be physically active, eat healthily, and have a positive body image, many other participants indicated their sexual orientation adversely impacted their exercise and eating behaviors. For example, some participants endorsed that there was greater body diversity in the LGBT community, while others reported that they felt like they needed their body to fit a particular aesthetic. Similarly, while approximately half of the participants denied that their sexual orientation posed any barriers to their physical activity, the other half of participants reported that they felt uncomfortable at the gym as a direct result of their sexual orientation. Finally, while many participants denied that their sexual orientation posed any impact on their eating behaviors, a substantial number of participants indicated that they engaged in binge eating due to their sexual orientation.

Additionally, research has identified specific protective factors for the LGBT population against disordered eating behaviors, including social support, being in a stable relationship, masculinity (regardless of biological sex), and self-compassion [37, 38, 120, 135, 153].

Overall, research demonstrates that individuals from the LGBT population may be at increased risk for clinical eating disorders and disordered eating behaviors, however certain protective factors exist as well. In order to fully elucidate the factors that affect LGBT individuals, it is important to recognize that not all minority groups are affected equally; it is likely that there are unique risk factors for individuals in each subgroup. The remainder of this review will present research regarding eating disorders and disordered eating behaviors in each LGBT subgroup (i.e., lesbian adults and adolescents, gay adults and adolescents, bisexual, mostly heterosexual, and questioning adults and adolescents, and transgender and gender non-conforming adults and adolescents). Furthermore, a discussion of proximal (i.e., direct) and distal (i.e., indirect) risk factors for eating disorders and disordered eating behaviors will be presented for each LGBT subgroup. Specifically, body dissatisfaction is a significant proximal risk factor for both eating disorders and disordered eating behaviors; the distal risk factors discussed below contribute indirectly to an increased risk of eating disorders and/or disordered eating behaviors by predicting increased body dissatisfaction, which, in turn, then contributes to eating disorders and/or disordered eating behaviors.

## Eating disorders and disordered eating behaviors within LGBT subgroups

### Lesbian adults and adolescents

Of all the LGBT subgroups, research findings regarding the rates of eating disorders and disordered eating behaviors among lesbian adults and adolescents are the least consistent [9].

Some of the literature suggests that adult and adolescent lesbians are at greater risk for clinical eating disorders and disordered eating behaviors. In a study conducted by Bell et al. [14], it was found that 34.7% of lesbian adults were currently or had previously been diagnosed with a full-syndrome eating disorder, and that an additional 66.7% reported significant clinical risk factors in which they were likely to develop an eating disorder. Similarly, adult lesbians also had more frequent clinical diagnoses of binge eating disorder than heterosexual women [74].

Researchers have found that adult lesbians appeared to diet frequently and exercised more than other groups [75, 139]. Further, adult and adolescent lesbians were also found to have significantly higher incidences of disordered eating behaviors than heterosexual women and men [42, 67, 88], reported more frequent binge eating, purging, and laxative use than heterosexuals, with binge eating occurring at higher rates than any other sexual orientation group [7, 31, 161]. Moreover, results of a longitudinal study revealed adult and adolescent sexual minority women were more likely to engage in restrictive dieting than heterosexual women [103].

Additionally, while other adolescents (e.g., heterosexuals, gay males) were found to decrease their disordered eating behaviors over time, the same was not true with adolescent lesbians. Indeed, the odds of fasting, using diet pills, and purging increased to being at least twice as likely at the end of their time in high school as compared to the beginning of their time in high school [167]. The findings from longitudinal research indicated that there was a greater disparity over time in fasting to lose weight for women with same-sex partners than for women with opposite-sex partners [168].

However, other studies have suggested that there are no significant differences between lesbian and heterosexual women in regard to eating disorder prevalence [53, 74, 131], nor for disordered eating behaviors and dieting behaviors [56, 140, 141, 172]. Indeed, some studies appeared to find more similarities than differences between lesbian and heterosexual females regarding rates of disordered eating behaviors [125, 172].

While some research suggests that lesbian adults may not be protected from eating pathology or body dissatisfaction [14, 126], other researchers have argued that, on average, adult and adolescent lesbians appear to be at less risk for eating disorders [143, 151]. Some findings suggested that they also report less engagement in disordered eating behaviors, including weight control methods such as dietary restriction and purging, dieting, and bingeing [99, 125, 134, 151, 162]. Additionally, some researchers found that in comparison to heterosexual and bisexual women, adult lesbians were more likely to report engaging in healthy eating behaviors and physical activity [159].

#### Proximal and distal risk factors

One study found that 82% of the lesbian participants based their self-worth upon their weight and 62.5% reported dissatisfaction with their eating patterns [14]. Additionally, studies have revealed that adult lesbians had lower self-esteem and greater feelings of ineffectiveness, interpersonal distrust, and difficulties identifying their emotions than heterosexual women did. Further, in comparison to heterosexual women, self-esteem in lesbians was found to be more dependent upon body esteem and BMI, suggesting that lesbians are vulnerable to societal pressure of the thin ideal [74, 75, 150, 172].

Furthermore, lesbian adults reported dissatisfaction with their weight [126] and exhibited greater body dissatisfaction than heterosexual women [88]. Lesbian adults also report higher levels of self-objectification compared to gay men and heterosexual men (but lower levels than heterosexual women [138]).

In a qualitative study conducted by Huxley et al. [83], many lesbian adults reported that male friends made negative comments about their weight; despite the women’s recognition of heterosexism in these comments, the effect was still hurtful. Moreover, none of the women reported increased body satisfaction after coming out about their sexual orientation, and some felt pressure from the LGBT community to be thin. The authors reported that “the lesbian women (many of whom were currently involved in LGB communities) argued that sexuality is irrelevant and stated that they did not feel ‘protected’ from social pressures because of an affiliation to lesbian subculture” ([83], p. 17). Similarly, further research found that the extent to which lesbians identified with their sexual orientation did not appear to influence body satisfaction or disordered eating behaviors [162].

It has also been found that adult and adolescent lesbians were more likely to report higher BMIs [18, 19, 88, 100, 117, 118], which in turn increased their risk for disordered eating behaviors [46, 68, 105]. These rates were predicted by age, education level, depression, public identification as a lesbian, increased heavy alcohol use, longer relationship length, lower relationship consensus, and the tendency to use eating as a coping mechanism in response to stress [106, 107, 112, 164].

However, it should be noted some research findings suggested no significant differences between lesbian and heterosexual women in body dissatisfaction, attitudes regarding weight and appearance, awareness of cultural standards of attractiveness, drive for thinness, likelihood of having a higher BMI, and body esteem concerning weight and physical appearance [15, 139, 140, 150, 172]. And, other investigators found no apparent differences in the path from internalization of the thin ideal to disordered eating behaviors [125, 172].

Some researchers have found that lesbians actually experienced lower body dissatisfaction and more body satisfaction, as further evidenced by their increased likelihood to have weighed significantly more and had a higher ideal weight reported greater body esteem concerning sexual attractiveness and were more satisfied with their weight and physical appearance (assuming “normal” BMI), expressed less concern regarding their weight and physical appearance, were less concerned with attempting to look like women in the media, and had a lower drive for thinness than adult and adolescent heterosexual females, which in turn reduced their likelihood of developing clinical eating disorders [3, 4, 6, 56, 67, 78, 99, 104, 125, 134, 140, 151, 162]. In contrast to the findings of Huxley et al. [83], other authors theorized that lesbians experienced less body dissatisfaction and were less vulnerable to eating disorders because they did not hold physical attraction and thinness as important as heterosexual women did, as they were not attempting to attract men [143]. However, it should be noted that one possible explanation for the discrepancy in findings could be the time when the research was conducted. Indeed, it appears that more recent studies find greater rates of disordered eating behaviors and body dissatisfaction in lesbian adults; these studies are more likely to have a larger and more diverse sample of lesbian adults, given that more individuals are open about their LGBT-identified status.

Risk factors related to sexual orientation included less time out about sexual orientation, less connection to the LGB community, low sexual identity development, and perceived stigma [14, 89]. Risk factors related to relationship dynamics included low social support and having an unmet need to belong [14, 89]. Risk factors related to mental health included depression, anxiety, and negative affect [14, 54, 89]. Risk factors related to demographics included being of Hispanic/Latina or black ethnicity [53], and risk factors related to intrapsychic functioning included low self-esteem [89]. Risk factors related to body image included body preoccupation, increased importance of fitness, and increased importance of being attractive [89]. It should be noted that no risk factors for eating disorders were found for adolescent lesbians; additional research in this area is needed.

Further distal risk factors have been identified that lead to disordered eating behaviors in adult and adolescent lesbians. For adult lesbians, risk factors related to sexual orientation included discrimination, concealment of sexual orientation, less involvement in the LGB community, internalized homophobia, internalized homonegativity, heterosexist experiences, proximal minority stress, lower sense of belonging to the lesbian community, organizations, and friends, and stigma consciousness [69, 70, 74, 107–109, 165]. Risk factors related to relationship dynamics included pressure from female partners to be thin, pressure from family to be thin, pressure from LGB friends to be thin, less social support from family, less social support from friends, less enjoyment of sexualization (i.e., enjoying positive sexualized male attention and feeling beautiful and sexy), and isolation [50, 83, 89, 107]. Risk factors related to mental health included anxiety, social anxiety, depression, negative affect, and eating as negative affect regulation [70, 74, 89, 107, 108, 110]. Risk factors related to demographics included being of an older age and being of Caucasian ethnicity [40, 74, 88, 131, 147]. Risk factors related to gender attitudes included negative femininity, low endorsement of women’s movement, less active work to improve the status of women, acceptance of traditional gender roles, realization of sexism, body-gender identity incongruence, lower masculinity, and non-identification as a feminist [40, 65, 77, 99]. Risk factors related to intrapsychic functioning included low self-esteem, reduced self-awareness, shame, interoceptive awareness, emotional control, self-blame, catastrophizing, and media internalization [12, 15, 69, 74, 77, 89, 97, 107, 109, 110, 131]. Risk factors related to body image included actual to ideal weight discrepancy, internalized sociocultural standards of beauty (i.e., media pressure to be thin, thin ideal internalization, internalized cultural attitudes concerning thinness), body esteem concerning weight, weight discrimination, physical condition, sexual attractiveness, sexual objectification, self-objectification, body surveillance, negative eating attitudes, and higher perceived weight status [40, 50, 69, 74, 77, 83, 88, 97, 103, 110, 165]. These risk factors predicted disordered eating behaviors directly, and indirectly via body dissatisfaction.

Additional studies identified more specific pathways linking specific risk factors to disordered eating behaviors among adult lesbians. Research conducted by Mason and Lewis [108] found that discrimination increased sexual minority stress, which in turn increased social anxiety, body shame, and thus binge eating. In addition, discrimination also was associated with greater negative emotions, which reduced self-awareness, resulting in increased binge eating [109]. Furthermore, lacking social support from family and friends was found to increase negative emotions and social anxiety, which then increased disordered eating behaviors, as did discrepancy between ideal weight and actual weight [110]. Older age and having a higher BMI predicted having a greater weight discrepancy, which in turn was associated with binging, purging, drive for thinness, and body dissatisfaction [40]. Moreover, it was found that body surveillance increased shame and negative eating attitudes, increasing disordered eating behaviors, which was further aggravated by internalized heterosexism [69]. Similarly, internalized homonegativity increased body surveillance, which increased body shame, and, ultimately, disordered eating behaviors [165].

A systematic review conducted by Mason et al. [111] that assessed for disordered eating patterns among sexual minority women identified themes among the current research studies, and proposed a model to predict eating disorder behaviors among sexual minority women. Specifically, this model indicated that gender experiences (i.e., gender roles, gender expression, sexual objectification, sexism/harassment), sexual orientation experiences (heterosexism, internalized minority stress, concealment, sexual identity), and the interaction of the two experiences affect the internalization of sociocultural norms in addition to social resources and emotion regulation, which in turn contribute to negative affect in addition to body image concerns and body surveillance, which ultimately predicted disordered eating behaviors.

Distal risk factors for disordered eating behaviors in adolescent lesbians include earlier age of achievement of sexual minority developmental milestones, depression, anxiety, and excessive alcohol use [35, 92]. These risk factors predicted disordered eating behaviors directly and indirectly via body dissatisfaction. See Table 1 for an overview of the risk factors for lesbian adults and adolescents.

**Table 1:** Eating Disorder and Disordered Eating Behavior Risk Factors in Lesbian Adults and Adolescents

Risk Factors	Eating Disorders	Disordered Eating Behaviors	
	Adults	Adults	Adolescents
Sexual Orientation	Less time out about sexual orientation Less connection to the LGB community Low sexual identity development Perceived stigma	Discrimination Concealment of sexual orientation Less involvement in the LGB community Internalized homophobia Internalized homonegativity Heterosexist experiences Proximal minority stress Lower sense of belonging to the lesbian community, organizations, and friends Stigma consciousness	Achieving sexual minority developmental milestones at a younger age
Relationship Dynamic	Low social support Unmet need to belong	Greater pressure from female partners Pressure from family Greater pressure from LGB friends Less social support from family Less social support from friends Less enjoyment of sexualization Isolation	
Body Image	Body preoccupation Greater importance of fitness Importance of being attractive Body image dissatisfaction Higher BMI	Actual to ideal weight discrepancy Internalized sociocultural standards of beauty Media pressure to be thin Thin ideal internalization Weight discrimination Internalized cultural attitudes concerning thinness Body esteem concerning weight Physical condition Sexual attractiveness Sexual objectification Self-objectification Body surveillance Negative eating attitudes Higher perceived weight status Body image dissatisfaction Higher BMI	Body image dissatisfaction Higher BMI
Intrapsychic Functioning	Low self-esteem	Low self-esteem Negative affect Reduced self-awareness Shame Internalization of sociocultural standards Interoceptive awareness Emotional control Self-blame Catastrophizing Media internalization	
Demographic	Hispanic/Latina or black ethnicity	Caucasian ethnicity Older age	
Mental Health	Depression Anxiety Negative affect	Social anxiety Depression Anxiety Eating as negative affect regulation	Depression Anxiety Excessive alcohol use
Gender Attitude		Negative femininity Low endorsement of women’s movement Less work for women’s status Acceptance of traditional gender roles Realization of sexism Body-gender identity incongruence Lower masculinity Non-identification as a feminist	

### Gay adults and adolescents

Overall, research has indicated that both adult and adolescent gay males were more likely to suffer from clinical eating disorders or report disordered eating behaviors compared to heterosexual males, with little variance in the studies. For example, in their study, Bell et al. [14] found that 14% of their sample of gay adults reported that they currently or previously suffered from an eating disorder, which is a significantly higher frequency than heterosexual men [81]. An additional one-half of their gay participants reported significant clinical risk factors in which they were likely to develop an eating disorder. Other studies also found gay adults to be at a higher risk for being diagnosed with an eating disorder than their heterosexual counterparts [44, 53, 73].

Early research findings suggest that gay adults reported more frequent dieting and greater dietary restraint, more binge eating, less control over their eating behaviors, more purging, and more exercise than heterosexual men [56, 99, 139] and these findings are supported by more contemporary research. Compared to heterosexual men, gay adults reported increased rates of binge eating, disordered eating behaviors, unhealthy weight control behaviors, food addiction, and diagnosed clinical eating disorders, in addition to poorer physical activity ([10, 20, 27, 54, 58, 67, 113, 127, 137, 141, 145, 146, 149, 152, 159, 161, 172, 173]).

Further, it was found that in comparison to their heterosexual counterparts, gay young adult and adolescent males were more likely to engage in exercising with intention to lose weight, restrictive eating, fasting, bingeing, purging, and use of diet pills, putting them at an increased risk for eating disorders [6, 7, 31, 167, 168, 174]. Additionally, it was found that they were less likely to attempt to gain weight, experienced a decrease in BMI from adolescence to early adulthood, and were less likely to engage in physical activity or team sports [33, 91, 117, 118].

Despite the findings reported above, Picot [131] reported that there does appear to be some variation in research results regarding the rates of eating disorders and disordered eating behaviors compared to their heterosexual counterparts. Furthermore, sexual minority male adolescents reportedly were likely to improve their disordered eating behaviors over time [154, 167].

#### Proximal and distal risk factors

The results discussed above are further supported by findings of greater body dissatisfaction, (i.e., poor body image, body image anxiety, drive for thinness, drive for muscularity, shape concerns, weight concerns), sociocultural influence (i.e., internalization of the thin ideal, susceptibility to advertising on physical appearances), eating concerns, frequency of engaging in conversations about appearances, and appearance orientation in gay adults compared to heterosexual men [2, 15, 36, 56, 58, 85, 99, 100, 126, 146, 172, 173]. Additionally, in one study, 63% of the gay participants reported basing their self-worth on their weight status, in addition to approximately one-half experiencing dissatisfaction with their eating patterns [14].

Furthermore, for gay adults, the discrepancy between current body shape and the body shape they believed they should have to attract a partner was significantly greater than their current body shape and ideal body shape. This discrepancy is associated with greater eating, shape, and weight concerns, suggesting that beliefs of partner body image preferences contribute to disordered eating behaviors in gay adults [57].

Additionally, it was found that in comparison to their heterosexual counterparts, gay young adult and adolescent males reported greater body dissatisfaction, reported greater desire for toned muscles, experienced a greater increase in weight and shape concern over time, were more concerned with trying to look like men in the media, and were more focused on being lean [6, 31, 33, 34]. Increased pornography use has been found to be associated with greater body dissatisfaction, drive for muscularity, increased eating disorder symptoms, and increased desire to use anabolic steroids in gay males [63].

Moreover, as previously discussed, being of a higher BMI is associated with increased disordered eating behaviors in adults [46, 68]. For gay men, having a higher BMI, experiencing more peer pressure, and lower levels of masculinity were associated with increased body dissatisfaction, which, in turn, was associated with greater disordered eating behaviors [80]. Having a higher BMI was predicted by age, employment status, depression, anxiety, and stress level [164].

Investigators have theorized that gay adults were less satisfied with their bodies and thus were more vulnerable to disordered eating behaviors due to the importance of physical attraction, and by extension thinness, in order to attract men via intrasexual competition [102, 143]. In a qualitative study conducted by VanKim et al. [160], gay adults reported feeling pressure to conform to the particular physical aesthetic ascribed to gay adults, which was associated with needing to be viewed as sexually attractive to other gay adults. Additionally, many described this ideal body shape as both muscular and thin, noting that thinness was unique to the gay male community (in comparison to heterosexual men), and that their masculinity influenced their body image and weight-related behaviors.

In an additional theory, it was hypothesized that because gay adults experienced greater levels of body shame and body objectification than heterosexual men, this, in turn, predicted increased rates of eating disorder symptomology among gay adults [104]. However, other research found that gay adults did not significantly differ from heterosexual men in terms of body esteem, body dissatisfaction, ideal body image, body image distortion, and drive for thinness [71, 131, 173].

Research has identified additional distal risk factors that may contribute to making gay adults particularly vulnerable to eating disorders. Specifically, risk factors related to sexual orientation included ambivalence about their sexual orientation, concern about the perception of others regarding their sexual orientation, attending a gay recreational group, pornography viewing, and sexual objectification experiences [52, 63, 142, 170]. Risk factors related to relationship dynamics included social media use [64]. Risk factors related to mental health included anxiety, depression, substance use disorder, specific phobia, diagnosis of any other psychiatric disorder, and childhood sexual abuse [52, 142]. Risk factors related to demographics included being of Latino/Hispanic or black ethnicity [53]. Risk factors related to gender attitudes included conforming to masculine norms and recalled childhood harassment for gender nonconformity [2, 170]. Risk factors related to body image included longer exercise sessions, internalization of cultural standards of attractiveness, body surveillance, steroid use, athletic appearance-ideal internalization, and upward appearance-based social comparisons [2, 62, 142, 170]. It should be noted that no empirical evidence of distal risk factors for eating disorders were found for adolescent gay males; more research in this area is needed.

Further distal risk factors were identified by research findings for disordered eating behaviors. For gay adults, risk factors related to sexual orientation included discrimination, concealment of sexual orientation, rumination on discriminatory experiences, internalized homophobia, internalized homonegativity, gay community identification (for thinner men), and belonging to the gay community [10, 23, 47, 82, 96, 136, 156, 163]. Risk factors related to relationship dynamics included lower relationship satisfaction, high-risk sexual behaviors, having an unmet need to belong, social sensitivity, being single, and frequency of usage of mobile dating apps [14, 17, 27, 29, 43, 124]. Risk factors related to mental health included childhood sexual abuse, negative affect, depression, and alcohol abuse [17, 24, 43]. Risk factors related to demographics included being of Caucasian ethnicity, being of an older age, and, conversely, being of a younger age [24, 131, 142]. Factors related to gender attitudes included negative femininity, gender role conflict, and greater levels of femininity [17, 99, 131]. Factors related to intrapsychic functioning included susceptibility to social messages, low self-esteem, low self-compassion, and media internalization[14, 15, 36, 43, 58, 82, 96, 131, 136, 156]. Factors related to body image included awareness of sociocultural norms regarding weight, implicit and explicit attitudes regarding weight, external motivation for working out, engaging in behaviors to increase muscle mass, pressure to diet, and body image disturbance [10, 15, 24, 43, 49, 142, 146, 156].

More specific pathways to disordered eating behaviors have been identified in the literature. It was found that an unmet need to belong and perceived stigma were predictive of increased depression and decreased self-compassion, which in turn were associated with higher levels of disordered eating behaviors among gay adults [14]. Moreover, gender role conflict (i.e., the incongruence between societal messages about gender norms and beliefs about what is achievable, resulting in psychological distress) was associated with negative affect and social sensitivity, which in turn were associated with body dissatisfaction and disordered eating behaviors [17].

Distal risk factors for disordered eating behaviors identified among gay male adolescents included earlier age of achievement of sexual minority developmental milestones, bullying, lack of support from adults, being of an older age, and lack of engagement physical activity [33, 92, 133]. These risk factors were found to be both directly predictive of disordered eating, and indirectly via body dissatisfaction. Table 2 provides an overview of the risk factors for gay adults and adolescents.

**Table 2:** Eating Disorder and Disordered Eating Behavior Risk Factors in Gay Male Adults and Adolescents

Risk Factors	Eating Disorders	Disordered Eating Behaviors	
	Adults	Adults	Adolescents
Sexual Orientation	Ambivalence about sexual orientation Concern about the perception of others regarding sexual orientation Attending a gay recreational group Sexual objectification experiences Pornography viewing	Discrimination Concealment of sexual orientation Rumination Internalized homophobia Internalized homonegativity Gay community identification (thinner men) Belonging to the gay community High-risk sexual behaviors	Achieving sexual minority developmental milestones at a younger age
Relationship Dynamic	Social media use	Lower relationship satisfaction Being single Unmet need to belong Social sensitivity Dating app usage	Bullying Lack of support from adults
Body Image	Longer exercise sessions Internalization of cultural standards of attractiveness Body surveillance Steroid use Athletic appearance ideal internalization Upward appearance-base social comparisons Body image dissatisfaction Higher BMI	Awareness of sociocultural norms regarding weight Implicit and explicit attitudes regarding weight External motivation for working out Engaging in behaviors to increase muscle mass Pressure to diet Body image disturbance Body image dissatisfaction Higher BMI	Lack of physical activity Body image dissatisfaction Higher BMI
Intrapsychic Functioning		Susceptibility to social messages Low self-esteem Low self-compassion Media internalization	
Demographic	Latino/Hispanic or black ethnicity	Caucasian ethnicity Older age Younger age	Older age
Mental Health	Anxiety Depression Substance use disorder Specific phobia Any psychiatric disorder Childhood sexual abuse	History of childhood sexual abuse Depression Negative affect Alcohol abuse	
Gender Attitude	Conformity to masculine norms Recalled childhood harassment for gender nonconformity	Negative femininity Greater levels of femininity Gender role conflict	

### Bisexual adults and adolescents

It should be noted that because bisexuality is a relatively new area of study, the research on the topics of eating disorders and disordered eating behaviors is recent and relatively limited. It does appear that the research findings to date on bisexuality suggest that there is a higher incidence of disordered eating behaviors, and by extension, eating disorders among bisexual, mostly heterosexual, and questioning men and women (i.e., [73, 145]); however, there is some conflicting evidence that the rates might not differ from that of heterosexual women [53] .

Research findings indicated that bisexual adults tended to experience increased binge eating, purging, and other disordered eating behaviors in comparison to their heterosexual counterparts [6, 7, 31, 42]. Bisexual men have been found to be at higher risk for eating disorders compared to heterosexual men [73]. Further, adult and adolescent bisexual females reported greater frequencies of fasting, purging, diet pill usage, laxative usage, weight cycling, smoking with intention to lose weight, skipping meals, body dissatisfaction, and overall disordered eating, and were less likely to report engaging in healthy eating behaviors and physical activity in comparison to heterosexual females, although they were less likely to engage in over-exercising with intention of weight loss [6, 88, 100, 103, 134, 141, 159, 168, 174]. Bisexual females have also been found to report higher rates of eating pathology compared to lesbian and gay individuals [141, 145, 147]. Additionally, it has been found that, unlike their heterosexual peers whose disordered eating rates improved with time, female bisexual adolescents saw no such improvement in their fasting, purging, and diet pill usage [167].

Additionally, adolescent bisexual males were found to be more likely to fast, use diet pills, purge, engage in poor physical activity patterns, and experience weight and shape concern, in addition to being less likely to attempt to gain weight [33, 67, 100, 168]. Further, bisexual and mostly heterosexual men were more likely to engage in unhealthy weight control behaviors in comparison to heterosexual and gay men [159]. Conversely, other investigators also found no difference in the prevalence of disordered eating behaviors between bisexual and heterosexual adult women [53].

#### Proximal and distal risk factors

Results of studies show young adult and adolescent bisexuals to experience greater body dissatisfaction and weight and appearance concerns in comparison to heterosexuals, suggesting elevated risk for developing eating disorders [6, 31]. More specifically, adolescent bisexual males were found to be more likely to view themselves as “overweight” or “obese” despite having a “normal” BMI and desire more toned muscles [67, 100].

Several studies have found that bisexual adults and adolescents were at greater risk for having an higher BMI than their heterosexual peers [5, 18, 88, 91, 100, 117, 118], which in turn increased their risk for disordered eating behaviors [46, 68, 133]. Interestingly, investigators have also found that bisexual girls were more satisfied with their bodies, and less concerned with attempting to look like women in the media, contrary to other findings [6].

For bisexual men, distal risk factors for eating disorders related to sexual orientation included attending a gay recreational group, ambivalence regarding their sexual orientation, concern about perception of others regarding their sexual orientation, gay community involvement, sexual objectification experiences, increased use of pornography, antibisexual discrimination, internalized biphobia, and sexual objectification experiences [26, 42, 52, 63, 170]. Risk factors related to relationship dynamics included social media use [64]. Risk factors related to mental health included anxiety, substance use disorder, specific phobia, diagnosis of any psychiatric disorder, and childhood sexual abuse [52]. Risk factors related to demographics included being of Latino/Hispanic or black ethnicity [53]. Risk factors related to gender attitudes included gender role orientation, recalled childhood harassment for gender nonconformity, and conformity to masculine norms [2, 42, 170]. Risk factors related to intrapsychic functioning included low self-esteem and maladaptive social comparison [42]. Risk factors related to body image included drive for muscularity, greater exercise frequency, internalization of cultural standards of attractiveness, body surveillance, steroid use, and upward appearance-based social comparisons [2, 26, 42, 62, 170].

For bisexual women, distal risk factors for eating disorders included gay community involvement, antibisexual discrimination, internalized biphobia, sexual objectification experiences, relationship dissatisfaction, depression, being of Latina/Hispanic or black ethnicity, gender role orientation, low self-esteem, maladaptive social comparison, objectified body consciousness, self-consciousness during physical intimacy, internalization of sociocultural standards of attractiveness, and body surveillance [26, 42, 53, 54, 142]. It should be noted that no empirical evidence to date has identified distal risk factors for eating disorders for bisexual adolescents; more research in this area is needed.

For bisexual men, distal risk factors for disordered eating behaviors related to sexual orientation included discrimination, concealment of sexual orientation, rumination about discrimination, internalized biphobia, internalized binegativity, gay community identification (for thinner men), and sexual objectification experiences [10, 23, 26, 47, 156, 163, 166]. Risk factors related to relationship dynamics included lower relationship satisfaction and being single [27, 29]. Risk factors related to mental health included childhood sexual abuse and depression [24, 154]. Risk factors related to demographics included being of an older age and being of Caucasian ethnicity [24, 142]. Risk factors related to intrapsychic functioning included greater susceptibility to social messages, low self-esteem, and reduced self-awareness [15, 58, 156]. Risk factors related to body image included awareness of sociocultural norms regarding weight, implicit and explicit attitudes regarding weight, external motivation for working out, engaging in behaviors to increase muscle mass, experiencing pressure to diet, the internalization of sociocultural standards of attractiveness, and body surveillance [10, 15, 24, 26, 142, 156]. These risk factors predicted disordered eating behaviors directly, and indirectly via body dissatisfaction.

For bisexual women, distal risk factors for disordered eating behaviors related to sexual orientation included discrimination, concealment of sexual orientation, sexual objectification experiences, internalized binegativity, internalized biphobia, and heterosexist experiences [26, 74, 165, 166]. Risk factors related to relationship dynamics included self-consciousness during physical intimacy, pressure from female partners, pressure from male partners, pressure from family, pressure from LGB friends, and peer appearance pressure [72, 83, 90]. Risk factors related to mental health included eating as negative affect regulation and depression [74, 154]. Risk factors related to demographics included being of an older age and being of Caucasian ethnicity [74, 88]. Risk factors related to intrapsychic functioning included low self-esteem and coping via internalization [12, 74, 166]. Risk factors related to body image included actual to ideal weight discrepancy, internalized sociocultural standards of beauty and attractiveness, media pressure to be thin, thin ideal internalization, higher perceived weight status, and body surveillance [26, 74, 83, 88, 90, 103, 165]. These risk factors predicted disordered eating behaviors directly, and indirectly via body dissatisfaction.

More specific pathways linking risk factors to disordered eating behaviors for bisexual adults have been identified in the literature. Antibisexual discrimination and internalized biphobia appeared to be associated with internalization of sociocultural standards of attractiveness, which increased body surveillance, sexual objectification, and body shame, which then predicted disordered eating behaviors among both men and women [26, 166]. Additionally, depression was found to be associated with disordered eating behaviors among bisexual adults, and, unlike their gay and lesbian counterparts, did not decrease with age [154]. Similarly, internalized homonegativity increased body surveillance, which increased body shame, and then disordered eating behaviors [165].

For bisexual adolescent males, distal risk factors for disordered eating behaviors included cyberbullying, lack of support from adults, being of an older age, being obese, and lack of engagement in physical activity [33, 133]. For bisexual female adolescents, distal risk factors included earlier age of achievement of sexual minority developmental milestones, bullying, depression, anxiety, and excessive alcohol use [35, 92]. These risk factors predicted disordered eating directly, as well as indirectly via body dissatisfaction. Refer to Tables 3 and 4 for an overview of risk factors for bisexual males and females, respectively.

**Table 3:** Eating Disorder and Disordered Eating Behavior Risk Factors in Male Bisexual Adults and Adolescents

Risk Factors	Eating Disorders	Disordered Eating Behaviors	
	Adults	Adults	Adolescents
Sexual Orientation	Attending a gay recreational group Ambivalence toward sexual orientation Concern about the perception of others regarding sexual orientation Gay community involvement Sexual objectification experiences Pornography viewing Antibisexual discrimination Internalized biphobia Sexual objectification experiences	Discrimination Concealment of sexual orientation Rumination Internalized biphobia Internalized binegativity Gay community identification (thinner men) Sexual objectification experiences	
Relationship Dynamic	Social media use	Being single Lower relationship satisfaction	Cyberbullying Lack of support from adults
Body Image	Maladaptive social comparison Drive for muscularity Exercise frequency Internalization of cultural standards of attractiveness Body surveillance Steroid use Upward appearance-based social comparisons Body image dissatisfaction Higher BMI	Awareness of sociocultural norms regarding weight Implicit and explicit attitudes regarding weight External motivation for working out Engaging in behaviors to increase muscle mass Pressure to diet Internalization of sociocultural standards of attractiveness Body surveillance Body image dissatisfaction Higher BMI	Lack of physical activity Body image dissatisfaction Higher BMI
Intrapsychic Functioning	Low self-esteem	Susceptibility to social messages Low self-esteem Reduced self-awareness	
Demographic	Latino/Hispanic or black ethnicity	Caucasian ethnicity Older age	Older age
Mental Health	Anxiety Substance use disorder Specific phobia Any psychiatric disorder History of childhood sexual abuse	History of childhood sexual abuse Depression	
Gender Attitude	Gender role orientation Recalled childhood harassment for gender nonconformity Conformity to masculine norms

**Table 4:** Eating Disorder and Disordered Eating Behavior Risk Factors in Female Bisexual Adults and Adolescents

Risk Factors	Eating Disorders	Disordered Eating Behaviors	
	Adults	Adults	Adolescents
Sexual Orientation	Gay community involvement Antibisexual discrimination Internalized biphobia Sexual objectification experiences	Discrimination Concealment of sexual orientation Sexual objectification experiences Internalized binegativity Internalized biphobia Heterosexist experiences	Earlier age of achievement of sexual minority development milestones
Relationship Dynamic	Relationship dissatisfaction	Self-consciousness during physical intimacy Pressure from female partners Pressure from male partners Pressure from family Pressure from LGB friends Peer appearance pressure	Bullying
Body Image	Maladaptive social comparison Objectified body consciousness Self-consciousness during physical intimacy Internalization of sociocultural standards of attractiveness Body surveillance Body dissatisfaction Higher BMI	Actual to ideal weight discrepancy Internalized sociocultural standards of beauty/attractiveness Media pressure to be thin Thin ideal internalization Higher perceived weight status Body surveillanceBody dissatisfaction Higher BMI	Body dissatisfaction Higher BMI
Intrapsychic Functioning	Low self-esteem	Low self-esteem Coping via internalization	
Demographic	Latina/Hispanic or black ethnicity	Caucasian ethnicity Older age	
Mental Health	Depression	Depression Eating as negative affect regulation	Depression Anxiety Alcohol abuse
Gender Attitude	Gender role orientation		

### Transgender and gender non-conforming adults and adolescents

While research on transgender and gender non-conforming individuals is in its infancy, it has been found that eating pathology is prevalent in this group [14, 128, 129] and that transgender and gender nonconforming adults experience eating disorders at a higher rate than their cisgender counterparts do [44, 145]. Further, the majority of studies assessing this population (i.e., [14, 45]) tend to group transgender and gender non-conforming individuals into one category; therefore, it is difficult to examine any potential differences in eating disorder and disordered eating behavior trends in the distinct populations.

Transgender adults and adolescents report higher incidences of fasting more than 24 h, laxative usage, diet pill usage, steroid usage without prescription, dietary restraint, bingeing, purging, and general disordered eating behaviors compared to cisgender peers [1, 51, 60, 66, 95, 128, 169]. In one study, almost 70% of the transgender and gender non-conforming adult participants reported dissatisfaction in their eating patterns, and 67.2% reported basing their self-worth on their weight status [14]. Transgender and gender non-conforming youth appear to be at particular risk for disordered eating behaviors [45]. For example, one study reported that transgender and gender non-conforming adolescents were more likely to be bullied for their weight or size and were less physically active compared to transgender youth [16].

Other studies have presented contradictory evidence, with findings that disordered eating among transgender individuals were either less than their cisgender counterparts (transgender males reported lower levels of bingeing and excessive exercise compared to cisgender males, and transgender females reported less excessive exercise than cisgender females [128]), comparable to that of their cisgender counterparts [93, 130], or were rarely experienced [170].

#### Proximal and distal risk factors

Body dissatisfaction appears to be a significant issue for transgender individuals [11], particularly for those who experience greater discrepancy between their external presentation and their internal identity [98]. One study [116] found that approximately 70% of their transgender participants (young adults and adolescents) experienced body dissatisfaction, well-documented as a proximal risk factor for eating disorders and disordered eating behaviors. Further, several studies found that transgender adults were more likely to report body dissatisfaction, and drive for thinness in comparison to their cisgender counterparts [66, 116, 171].

Distress regarding body parts related to an individual’s undesired assigned sex and body shame may be highly linked to body dissatisfaction for transgender individuals [13, 41], as well as feelings that a change in one’s body size could subsequently change levels of masculinity and/or femininity in one’s appearance [116].

Similarly, early research findings suggested that individuals assigned female at birth experiencing gender dysphoria were also more likely to report disordered eating behaviors [144]. Transgender women (those who were assigned male sex at birth but whose gender identify is female) may engage in dietary restriction in order to suppress characteristics related to their birth sex (e.g., larger body frame, muscles) or may strive for thinness in order to align more with their desired gender [1]. Additionally, they may attempt to align themselves with (or internalize) the thin-ideal for women presented in Western media [41, 171] and therefore may be at increased risk for disordered eating behaviors [87] compared to transgender men (individuals who were assigned female sex at birth but whose gender identify is male). Finally, one study found that transgender men appeared to be at a greater risk for higher BMIs [164], which is also associated with higher levels of disordered eating behaviors [46, 68]. Conversely, some research has found that body dissatisfaction among transgender individuals were either comparable to that of their cisgender counterparts [22, 93], or were rarely experienced [170]. Gender dysphoria (i.e., extreme distress resulting from the incongruence between one’s internal gender and sex assigned at birth) also appears to be a risk factor for disordered eating behaviors in transgender and gender non-conforming youth who may be more likely to engage in disordered eating behaviors in order to attempt to manipulate their body shape and size, as to feel more aligned with their authentic gender, while suppressing the secondary sex characteristics associated with their sex assigned at birth [123]. Additionally, eating disorder behaviors may be utilized to prevent puberty onset or progression of puberty [39]. Disordered eating behaviors are also more likely to be reported by individuals who encounter barriers to gender confirmation treatment, such as lack of parental consent and lack of timely referral to treatment [45]. Indeed, the experience of gender dysphoria appears to be a significant motivation, and thus a distal risk factor related to body dissatisfaction, for transgender individuals to develop eating disorders [148, 158].

Research findings have also identified distal risk factors that increase transgender individuals’ vulnerability to the development of clinical eating disorders. Specifically, risk factors for transgender adults included not being on HRT, non-affirmation of their gender identity, anxiety, perfectionism, low self-esteem, and identification as a sexual minority [86, 93, 145, 155]. For transgender adolescents, risk factors included lack of timely gender dysphoria management, suicidal ideation, suicide attempt, and self-injurious behaviors [45, 169].

Furthermore, additional research identified distal risk factors for disordered eating behaviors among transgender and gender non-conforming individuals. Risk factors for transgender adults included antitransgender discrimination, social distress, self-criticism, sexual objectification, internalization of sociocultural standards of attractiveness, and body surveillance [25, 116]. Risk factors for transgender adolescents included harassment by peers or school personnel, feeling less safe at school, discrimination, stigma, social distress, and self-criticism [115, 116, 132, 169]. These risk factors predicted disordered eating behaviors directly, as well as indirectly via body dissatisfaction.

Additional research indicated more specific complex pathways linking proximal and distal risk factors to disordered eating behaviors among transgender and gender non-conforming adults. McGuire et al. [116] found that self-criticism and social distress were associated with body dissatisfaction. Additionally, in accordance with the interpersonal theory of eating disorders, experiencing an unmet need to belong and perceived stigma were associated with less self-compassion, which then predicted likelihood of developing an eating disorder [14]. Furthermore, for transgender women in particular, in accordance with the objectification theory, experiencing dehumanization led to greater internalization of sociocultural standards of attractiveness, which in turn was associated with greater body surveillance, body dissatisfaction, and disordered eating behaviors [25]. See Table 5 for a summary of risk factors for transgender and gender non-conforming individuals.

**Table 5:** Eating Disorder and Disordered Eating Behavior Risk Factors in Transgender Adults and Adolescents

Risk Factors	Eating Disorders		Disordered Eating Behaviors	
	Adults	Adolescents	Adults	Adolescents
Gender Minority	Not being on HRT Non-affirmation	Lack of timely gender dysphoria management	Antitransgender discrimination	Harassment Discrimination Stigma
Relationship Dynamic			Social distress	Social distress Less safe at school
Body Image	Body image dissatisfaction Higher BMI	Body image dissatisfaction Higher BMI	Sexual objectification Internalization of sociocultural standards of attractiveness Body surveillance Body image dissatisfaction Higher BMI	Body image dissatisfaction Higher BMI
Intrapsychic Functioning	Perfectionism Low self-esteem		Self-criticism	Self-criticism
Mental Health	Anxiety	Suicidal ideation Suicide attempt Self-injurious behaviors		

## General conclusion

In congruence with Meyer’s sexual MSM (2003), and its expansion to include transgender individuals [76], the research findings reviewed here indicate that adults and adolescents in the LGBT population tend to be at greater risk for both subclinical disordered eating behaviors, as well as full-syndrome eating disorders in comparison to their heterosexual and cisgender counterparts. Several of the studies reviewed demonstrated that distal stressors, such as stigma and discrimination, and proximal stressors, such as internalized homophobia or transphobia and concealment of sexual or gender identity, were linked to increased risk of eating pathology (i.e., [14, 79, 135, 163]), which is consistent with general research that reports that that minority stress increased risk for the development of physical and mental health issues [26, 101, 118, 121, 122].

However, risk factors related to the MSM were not uniform across all subgroups. More specifically, having greater connection to other sexual minorities and being involved in the LGB community were found to be risk factors for eating pathology among gay and bisexual adults [47, 52, 96]. Conversely, having less connection to other sexual minorities and not belonging to the lesbian community were found to be risk factors for eating pathology among lesbian adults [70, 74, 89]. Further, regarding adolescents, the only subgroup that literature findings explicitly connect stigma and discrimination to increased disordered eating behaviors is that of transgender adolescents [169]. However, given the amount of evidence that stigma and discrimination are risk factors for each adult subgroup (i.e., [14, 108, 163]), it is likely that this same pattern is present in each adolescent subgroup and that more research is needed.

Examination of the risk factors that appear to contribute to higher frequencies of eating disorders and disorders eating behaviors among LGBT adults and adolescents demonstrate risk factors above and beyond those solely associated with the MSM. These additional risk factors are related to relationship dynamics, gender attitudes, body image, intrapsychic functioning, demographics, and mental health, which can contribute to risk or provide protection from eating pathology.

Patterns within specific risk factors were present across subgroups. Among adult subgroups, less connection to others (e.g., lack of support, unsatisfying relationships, lack of belonging, social media use) was common within relationship dynamics as a risk factor for eating disorders [14, 64, 89]. Body image dissatisfaction; higher BMI; greater importance of appearance; internalization of the ideal body; body surveillance, preoccupation, and consciousness; increased exercise; and higher levels of objectification were common within body image factors [2, 26, 42, 86, 89, 142, 170]. Low self-esteem was a common intrapsychic factor [42, 86, 89], aside from gay adults, as discussed below. Finally, mood disorders (e.g., depression), anxiety disorders (e.g., general, social), and other psychiatric conditions (e.g., phobias, substance use, trauma) were common within mental health factors [14, 52, 54, 86, 89, 142].

Similar patterns were found for disordered eating behaviors. Across adult subgroups, having less social support, feelings of not belonging, and isolation (e.g., being single, fear of intimacy, low relationship satisfaction) were common within relationship dynamic factors [14, 17, 27, 29, 43, 50, 83, 89, 90, 107]. Body image dissatisfaction, higher BMI and perceived weight status, internalization of the ideal body and cultural standards of attractiveness, pressure to be thin, and objectification were common within body image factors [10, 15, 24–26, 40, 43, 46, 49, 50, 68, 69, 74, 77, 83, 88, 90, 97, 103, 110, 142, 146, 156, 165]. Low self-esteem, self-criticism, and media internalization were common within intrapsychic factors [12, 14, 15, 36, 43, 58, 74, 82, 96, 131, 136, 156]. Mood disorders (e.g., depression), anxiety disorders (e.g., general, social), and other psychiatric conditions (e.g., phobias, substance use, trauma) were common within mental health factors [17, 24, 43, 70, 89, 107, 108, 110, 154]. Finally, negative femininity was common within gender attitude factors [99]. Although there is less research on risk factors among LGBT adolescents, some patterns still emerged across adolescent subgroups. Specifically, depression and anxiety were common within mental health risk factors [92].

In addition to the patterns of similarities, there were also differences in risk factors for eating disorders noted across subgroups. For example, low self-esteem was found to be an intrapsychic functioning risk factor across all subgroups except gay adults; however, this is likely due to lack of research, rather than lack of presence, especially since this was found to be a risk factor for disordered eating behaviors. Additionally, among sexual minority men, steroid use was found to be a risk factor for eating disorders [62]; this is likely only present in males due to pressure to conform to the muscular ideal. Further, sexual minority men possessed a unique gender attitudes risk factor of recalled memories about harassment for gender nonconformity during childhood [170].

Likewise, differences in risk factors for disordered eating behaviors were also present across the subgroups. For instance, peer pressure was a relationship dynamic risk factor present for solely sexual minority women [72, 83]; this risk factor is likely present among sexual minority men, especially considering is it part of the Tripartite Influence model of eating disorders, but is lacking research. Additionally, coping via internalization was found to be a unique intrapsychic functioning risk factor for bisexual women [166]. Further, for lesbian adults, not identifying as a feminist and the acceptance of traditional gender roles were found to be gender attitude risk factors [40, 65], as was less enjoyment of sexualization [50]. Conversely, among gay men, gender role conflict was found to be a risk factor for disordered eating behaviors [17]. Finally, among sexual minority men, the use of dating apps was found the be a relationship risk factor [124], which is likely due to the added pressure to adhere to a certain aesthetic to attract more potential sexual partners.

This research seems to indicate that for adults and adolescents from LGBT subgroups, individual risk factors in conjunction with the minority stress risk factors may account for increased risk of developing eating pathology compared to heterosexual and cisgender individuals.

### Limitations and future directions

Although this review increases current knowledge regarding the risk factors for the development of disordered eating behaviors and clinical eating disorders in sexual and gender minority individuals, it is not without limitations. First, we recommend that researchers conduct a systematic review or a meta-analysis in order to more fully elucidate the risk factors present in each subgroup. We also did not fully examine protective factors for each subgroup and recommend additional research examining both risk and protective factors for all subgroups, particularly lesbian adults and adolescents, given the contradictory findings in the literature. Also, as research on bisexual and transgender and gender non-conforming individuals is in its infancy, we recommend further examination of factors that may be most relevant to these groups. Notably, research findings on eating disorder risk factors among LGB adolescent subgroups were absent. Future research should assess risk factors for eating disorders unique to lesbian, gay, and bisexual adolescent groups. Similarly, research on risk factors for disordered eating behaviors among all adolescent subgroups was lacking in comparison to that of the adult subgroups; this issue would benefit from further research as well.

Further, this review did not adequately address the risk factor of race and ethnicity. Although research indicated that White/Caucasian ethnicity was a risk factor for disordered eating behaviors among the LGBT subgroups, it should also be recognized that historically, there has been a racial disparity in access to mental health care, with racial minorities being less likely to seek treatment for mental health (i.e., [114]). Therefore, future research should assess whether racial identity is truly a risk factor, or if the difference in rates of disordered eating behaviors may be due to lack of access to the necessary mental health care.

Finally, while sexual orientation and gender identity are related, they are two separate constructs that can individually increase the likelihood of exposure to stigmatization and discrimination [145], therefore it is important to consider the intersection between sexual and gender identity, as identification with more than one marginalized identity is linked to increased health risk [8]. Overall, additional research examining eating pathology within the MSM framework is warranted.

### Clinical implications

Due to the research findings indicating that adults and adolescents in the LGBT population tended to be at greater risk for both subclinical disordered eating behaviors, as well as full-syndrome eating disorders, it is of the utmost importance that clinicians working with individuals from this population thoroughly assess for disordered eating behaviors, in addition to any proximal and/or distal risk factors present for eating disorders and disordered eating behaviors. This is especially important given that this population is more likely to experience other distal and proximal stressors related to the MSM [121].

Given the high likelihood of experiencing stigma, it is crucial that clinicians are educated on how to respectfully interact with the LGBT population, as not doing so can further alienate LGBT individuals and be detrimental to treatment. Indeed, one study identified that transgender individuals in eating disorder treatment frequently reported deficits in their clinician’s gender competence, leading them to believe receiving treatment for their eating disorder was ineffective and harmful [48]. Similarly, another study identified specific improvements clinicians can implement when working with transgender individuals diagnosed with eating disorders, including using patients’ correct gender pronouns, receiving training in cultural and clinical competency in transgender health, and facilitating access to gender-affirming interventions [21].

It is also important for educators and school administrators to receive education regarding the risk factors for individuals within the LBGT community and to seek to provide an accepting environment for individuals from sexual and gender minority groups. It has been found that harassment from peers and school personnel is correlated with negative outcomes [115], therefore the importance of an accepting and diverse school environment cannot be understated. We also recommend that educators work to create policies that focus on the protection and acceptance of individuals from sexual and gender minority groups.

Additionally, past studies have identified specific components to include in interventions targeting the LGBT population, including nutritional counseling, media literacy, body image cognitive dissonance, avoiding negative body talk, and body activism, while attending to the particular vulnerabilities of the LGBT population (e.g., sexual and gender minority stress due to greater discrimination and stigma), and while affirming their sexual orientation and/or gender minority status [28, 55]. We recommend continued research examining interventions that utilize these components in order to best support individuals from the LGBT community. Although a focus on individual resilience and factors that can protect individuals is important, it is also vital to enhance community resilience (equipping the community to provide resources and support for sexual and gender minority individuals such as hotlines, support groups, role models, and policies and laws that advocate for LGBT individuals) as well [122].

Although the research over the past few decades has expanded our insight into eating disorders and disordered eating behaviors in the LGBT population, significant gaps in the literature remain. In order to best understand and treat this important issue, researchers and clinicians should endeavor to further examine the role of the MSM in the development of eating pathology in sexual and gender minority groups.

## Data Availability

All supporting data used in this literature review is referenced.

## Abbreviations

AN: Anorexia nervosa
BN: Bulimia nervosa
BED: Binge eating disorder
BMI: Body Mass Index
DEB: Disordered eating behaviors
ED: Eating disorders
HRT: Hormone-replacement therapy
LGBT: Lesbian, gay, bisexual, and transgender
MSM: Minority Stress Model
